# Effect of Pioglitazone on In-Stent Restenosis after Coronary Drug-Eluting Stent Implantation: A Meta-Analysis of Randomized Controlled Trials

**DOI:** 10.1371/journal.pone.0109614

**Published:** 2014-10-03

**Authors:** Ming-duo Zhang, Yu-hui Zhang, En-jun Zhu, Shi-bin Qiao, Shu-zheng Lv, Quan-ming Zhao

**Affiliations:** 1 Department of Cardiology, Beijing Anzhen Hospital, Capital Medical University, Beijing, China; 2 Beijing Institute of Heart, Lung and Blood Vessel Diseases, Beijing, China; 3 Department of Cardiac Surgery, Beijing Anzhen Hospital, Capital Medical University, Beijing, China; 4 Department of Cardiology, Rizhao People's Hospital, Shandong, China; University of Bologna, Italy

## Abstract

**Background:**

In-stent restenosis (ISR) remains a common life-threatening complication and some studies have shown that pioglitazone can reduce the incidence of ISR in patients with drug-eluting stents (DES) implantation. We conducted a meta-analysis to assess the effect of pioglitazone in preventing ISR after DES implantation.

**Methods:**

Randomized controlled trials (RCTs) investigating the effects of pioglitazone for ISR after DES implantation were identified by systematic searches of multiple online databases and manual searches of related reference lists of identified trials through May 2014. The primary endpoint was the rate of ISR. Secondary endpoints included minimum lumen diameter, percentage stenosis of stented vessels, late loss, in-stent neointimal volume, target vessel revascularization (TVR), target lesion revascularization, myocardial infarction, stent thrombosis and death.

**Results:**

Five studies, comprising 255 pioglitazone-treated patients and 245 controls, were identified in the current meta-analysis. Pioglitazone did not significantly reduce the rate of ISR (P = 0.20) with low heterogeneity (I^2^ = 13.3%, P = 0.32). For the secondary outcomes, pioglitazone did not substantially affect the pooled estimates of these endpoints except late loss (P = 0.01) and TVR (P = 0.04).

**Conclusions:**

The limited evidence indicates that pioglitazone does not demonstrate markedly beneficial effect in patients subjected to coronary DES implantation. However, the results should be interpreted with care given the small sample size. Further large-scale RCTs are needed.

## Introduction

In-stent restenosis (ISR), stenosis more than 50% at the site of stent [1], has been considered as the leading problem after percutaneous coronary intervention (PCI). A meta-analysis showed that drug-eluting stent (DES) compared with bare-metal stent (BMS) markedly reduced the incidence of ISR [2]. However, a fairly high rate of ISR (about 10%) after DES implantation still exists. Currently, no drug is in routine use other than dual antiplatelet therapy to prevent ISR. Many pharmacologic agents demonstrated efficacy in reducing restenosis after PTCA or BMS implantation [3], [4], [5], [6]. However, none of them has been performed with DES. Thiazolidinediones (TZDs), which are widely used as insulin-sensitizers in the treatment of diabetes mellitus [7], [8], can inhibit proliferation and migration of vascular smooth muscle cells (VSMCs) and reduce intimal proliferation after vascular injury [9], [10], [11], [12], [13]. These evidences provide the rationale for assessing effect of TZDs on limiting ISR.

Three TZDs have received approval for glycaemic control in type 2 diabetes mellitus (T2DM), troglitazone (withdrawn due to liver toxicity) [14], rosiglitazone and pioglitazone. Clinical studies have indicated that rosiglitazone is associated with adverse cardiovascular events [15]. On the contrary, pioglitazone shows beneficial effects on cardiovascular outcomes [16]. Thus, in this study, pioglitazone was chosen as the study drug.

Some randomized controlled trials (RCTs) [17], [18], [19], [20], [21], [22] and meta-analyses [23], [24], [25] have indicated that pioglitazone is effective in decreasing incidence of ISR after BMS implantation. Several small studies have investigated the efficacy of pioglitazone in the reduction of ISR after DES implantation [26], [27], [28], [29], [30]. However, results of these studies were inconsistent. Therefore, to determine whether pioglitazone can reduce the incidence of ISR, we performed this meta-analysis of related studies to investigate the effect of pioglitazone in preventing of ISR after DES implantation.

## Methods

This meta-analysis was written with reference to the PRISMA statement [31], and the PRISMA checklist is provided as Checklist S1.

### Data Sources and Searches

We searched for all RCTs that investigated the effects of pioglitazone for restenosis after DES implantation in PubMed (http://www.ncbi.nlm.nih.gov/pubmed) and EMBASE (http://www.embase.com). In addition, four Chinese databases, including CNKI (http://www.cnki.net), CBM (http://www.sinomed.ac.cn), Wanfang (http://www.wanfangdata.com.cn), and VIP (http://www.cqvip.com), were also retrieved (up to May 2014). Relevant articles were identified using the following Medical Subject Heading (MeSH) terms and keywords: ‘thiazolidinedion*’, ‘pioglitazon*’, ‘Peroxisome proliferator activated receptor gamma’, ‘atherosclerosis’, ‘coronary heart disease or CHD’, ‘coronary artery disease or CAD’, ‘ischemic heart disease or IHD’, ‘myocardial infarction or MI’, ‘stent*’ and ‘restenosis or restenoses’. We examined the references cited in the identified articles to include other potentially eligible studies. We also checked the reference lists of relevant review articles and journals. If several reports overlapped with each other, only the most detailed one was kept. The language of identified studies limited to Chinese or English. Studies included in the meta-analysis satisfied the following criteria: (i) randomized controlled trials were limited to human subjects; (ii) patients were individuals undergoing DES implantation, with or without diabetes mellitus; (iii) studies compared pioglitazone with placebo for restenosis after DES implantation; (iv) in addition to study medications, all patients received recommended post-PCI medical interventions such as aspirin, statins, beta blockers, angiotensin-converting enzyme inhibitor; (v) sufficient information was supplied for both baseline and follow-up angiography and/or intravascular ultrasound (IVUS) data; (vi) subjects were followed for at least 6 months.

### Outcome Measures

ISR as the primary outcome was measured by quantitative angiographic analysis (QCA). The secondary outcomes included: 1) minimum lumen diameter and percentage stenosis of stented vessels; 2) late loss (change in minimum lumen diameter at the stent site from baseline to follow-up; 3) in-stent neointimal volume measured by IVUS; 4) target lesion revascularization (TLR), target vessel revascularization (TVR), MI, stent thrombosis and death were also considered as secondary endpoints.

### Data Extraction and Quality Assessment

Data were independently extracted by 2 reviewers (Ming-duo Zhang and Yu-hui Zhang). Discrepancies about study inclusion between the two reviewers resolved by a consensus or a third review author(Quan-ming Zhao). We extracted the following information from each study: first author, publication year, number of cases and controls, the characteristics of subjects, interventions in each group (initial time, dosage and duration), stent type, study design, duration of follow up, results of QCA and/or IVUS, incidence of TLR and TVR, incidence of MI, stent thrombosis and death.

The quality of included studies was evaluated with the Jadad method [32]. This scale includes three subscales as follows: randomization (0–2 points), blinding (0–2 points), and dropouts and withdrawals (0–1 point). The quality scale ranges from 0 to 5 points. The studies were divided into low quality (score≤2) and high quality groups (score ≥3) [32], [33].

### Statistical Analysis

All statistical analyses were conducted with STATA 10.0 (Stata Corp., TX, USA). Odds ratios (ORs) and their 95% confidence intervals (CIs) were used to estimate the results of the dichotomous data, including ISR and clinical outcomes (TVR, TLR, MI, stent thrombosis, and death). Weighted mean difference (WMD) with their 95% CIs were calculated to evaluate continuous data obtained from QCA (minimal lumen diameter, late loss, and percentage stenosis) and IVUS studies (neointimal volume). Statistical heterogeneity among trials was assessed by the Cochran's Q test and considered significant for P<0.10 [34]. We also calculated the inconsistency index *I^2^* statistic to assess total variation across among studies that is caused by heterogeneity rather than chance [35]. *I^2^* was expressed as percentage and ranged from 0 to 100% (*I^2^*<25%, correspond to no or mild heterogeneity; 25% ≤ *I^2^*<50%, correspond to moderate heterogeneity; 50% ≤ *I^2^*<75%, correspond to large heterogeneity; 75% ≤ *I^2^*, correspond to extreme heterogeneity). We used the random-effects model described by DerSimonian and Laird [36] to calculate pooled estimates and the significance of the pooled estimates was determined using a *Z*-test. We performed sensitivity analyses to assess the robustness of our results. One method was influential analysis, which was performed by excluding one study each time and determined whether any single study could alter the overall pooled estimate. Another method was to examine whether the ORs are significantly changed when we removed studies according to the following prespecified variables: (1) with or without DM; (2) duration of follow-up; (3) dosage of intervention medication. Assessment of publication bias was performed using a modified funnel diagram [37]. An asymmetric diagram suggests potential this bias. The asymmetry was evaluated by the Begg and Mazumdar's rank correlation method [37] and Egger's linear regression method [38]. All P values were two-sided, and P values less than 0.05 were considered to indicate statistical significance.

## Results

### Identification and Selection of Eligible Studies

**Figure 1:**
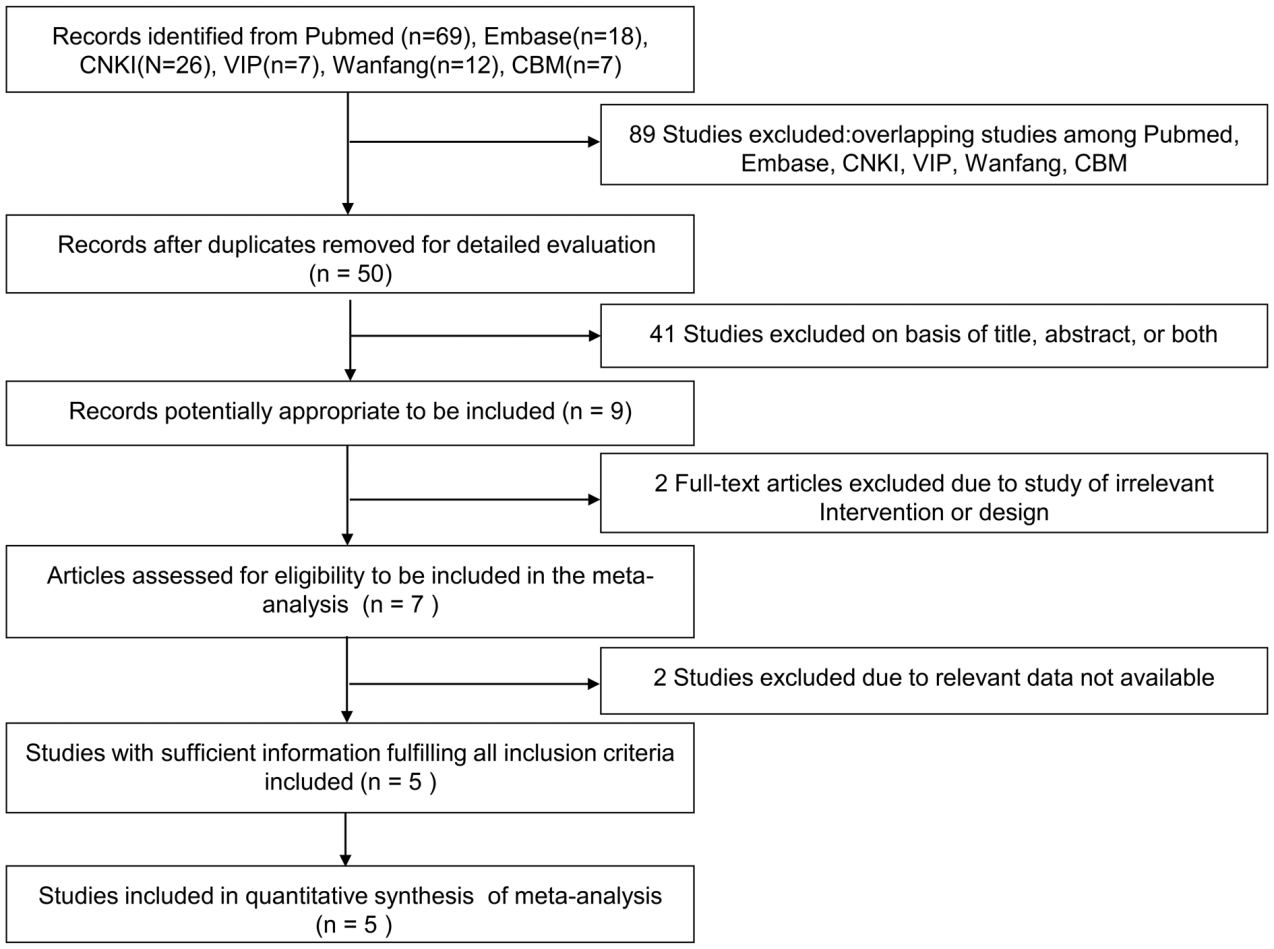
Flow diagram of the study selection.

After a detailed review of retrieved articles, 139 potentially related articles were identified in the initial analysis. Eighty-nine studies were eliminated due to duplication, and 41 studies were eliminated on basis of titles and/or abstracts. Two full text articles excluded due to study of irrelevant intervention or design and 2 studies excluded due to relevant data were not available. Finally, five relevant RCTs comprising 255 cases and 245 controls were included in the current meta-analysis [26], [27], [28], [29], [30]. The process of selecting studies for the meta-analysis can be seen in Figure 1.

### Characteristics of the Included Studies

All the included studies were published between 2009 and 2013. All RCTs had at least 6-month follow-up angiography as planned [26], [27], [28], [29], [30]. The sample size in these studies ranged from 60 to 128 (total, 500). All the patients of included studies receiving DES. Two studies only used first-generation sirolimus-eluting stents [26], [27]. Two studies only used second-generation zotarolimus-eluting stents [28], [30]. One study used both [29]. Individual of three studies received 15-mg of pioglitazone each day [26], [28], [29] and the other studies were given 30-mg once-daily [27], [30]. Among all the 5 studies included, three studies reported ISR [27], [29], [30] and percentage stenosis of stented vessels [26], [29], [30], four reported minimum lumen diameter and late loss [26], [28], [29], [30] and two reported neointimal volume [29], [30]. The quality of the included studies in the current meta-analysis was evaluated using Jadad's scale. The mean Jadad score of included studies was 3.2. The detailed characteristics of the included trials are described in Table 1 and 2.

**Table 1:** Main Characteristics of the Studies Included in the Meta-Analysis.

Authors/Year	Study Population	Study Type	Stent Type	FU, m	Jadad Score	Sample Size, n	Age, years	Male gender, n	Smoker, n	BMI	Intervention
Li et al./2009	Non-DM	RCT	ZES	6	2	PIO: 46	70±12	NR*	41	25.3±2.2	15 mg/day, after PCI
						CONT: 51	69±13	NR*	44	25.1±2.3	Placebo
Hong et al./2010	T2DM	RCT	ZES	8	4	PIO: 47	63.5±7.4	32	10	23.4±2.4	30 mg/day, after PCI
						CONT: 47	62.4±8.3	29	12	23.2±4.3	Placebo
Wei et al./2012	Non-DM	RCT	SES	6	4	PIO: 71	60.38±8.93	48	NR*	NR*	30 mg/day, after PCI
						CONT: 57	61.19±9.36	43	NR*	NR*	Placebo
Lee et al./2013	T2DM	RCT	Mixed†	12	3	PIO: 60	60.3±9.53	43	30	24.0±2.96	15 mg/day, after PCI
						CONT: 61	61.9±8.75	46	30	23.8±3.23	Placebo
Zhang et al./2013	Non-DM	RCT	SES	6	3	PIO: 31	NR*	NR*	NR*	NR*	15 mg/day, after PCI
						CONT: 29	NR*	NR*	NR*	NR*	Placebo

BMI, Body Mass Index; CONT, control; DES, Drug Eluting Stent; DM, Diabetes Mellitus; FU, Follow-Up; NR, Not Recorded; RCT, Randomized Controlled Trial; SES, Sirolimus-Eluting Stents; PCI, Percutaneous Coronary Intervention; PIO, Pioglitazone; ZES, Zotarolimus-Eluting Stents.

*Data was not supplied but similar between groups.

† both stent types were used.

**Table 2:** Angiographic, IVUS and Clinical Events Data.

Authors/Year	QCA data					IVUS data		Clinical Events					
	Patients, n	ISR(%)	MLD (mm)	LL(mm)	PS(%)	Patients, n	Neointimal volume (mm^3^)	Patients, n	TVR	TLR	MI	Stent Thrombosis	Death
Li et al./2009	PIO: 46	NR	1.52±0.97	0.98±0.93	NR	NR	NR	PIO: 46	3	NR	5	NR	0
	CONT: 51	NR	0.76±0.69	1.91±0.89	NR	NR	NR	CONT: 51	11	NR	13	NR	2
Hong et al./2010	PIO: 40	6 (15.0)	2.30±0.41	0.41±0.40	20±14	PIO: 47	1.3±0.7	PIO: 40	0	0	0	NR	0
	CONT: 38	8 (21.1)	2.09±0.53	0.65±0.54	28±17	CONT: 47	2.5±1.4	CONT: 38	0	0	0	NR	0
Wei et al./2012	PIO: 71	2(2.82)	NR	NR	NR	NR	NR	PIO: 71	0	0	0	0	0
	CONT: 57	7(12.28)	NR	NR	NR	NR	NR	CONT: 57	0	0	0	0	0
Lee et al./2013	PIO: 51	4 (9.3)	2.25±0.55	0.35±0.57	20.00±13.50	PIO: 18	1.86±1.04	PIO: 51	0	6	2	1	0
	CONT: 54	4 (7.5)	2.35±0.59	0.31±0.60	18.46±16.78	CONT: 16	2.08±0.58	CONT: 54	0	6	1	2	1
Zhang et al./2013	PIO: 31	NR	2.75±0.43	0.21±0.17	7.95±6.72	NR	NR	PIO: 31	0	0	0	0	0
	CONT: 29	NR	2.35±0.55	0.62±0.20	19.95±8.31	NR	NR	CONT: 29	0	0	0	0	0

CONT, Control; ISR, In-Stent Restenosis; IVUS, intravascular ultrasound; LL, Late Loss; MI, Myocardial Infarction; MLD, Minimum Lumen Diameter; NR, Not Recorded; PIO, Pioglitazone; PS, Percentage Stenosis; TLR, Target Lesion Revascularization; TVR, Target Vessel Revascularization; QCA, Quantitative Coronary Angiography.

### The Primary Outcome: ISR

**Figure 2:**
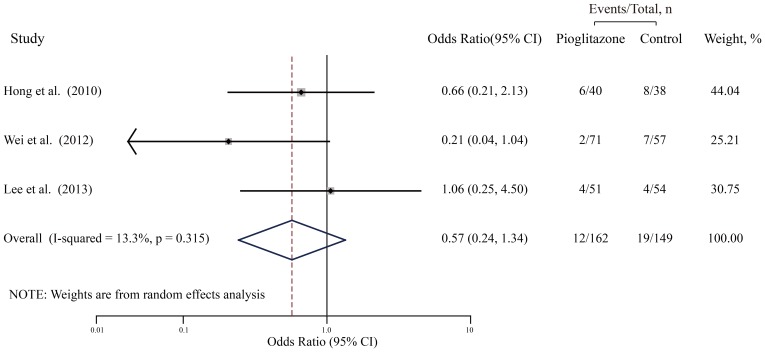
Meta-analysis of studies assessing effects of pioglitazone on the incidence of ISR. Black boxes indicate the odds ratios (ORs) with horizontal lines representing 95% CI (confidence interval). Hollow diamond plot indicates the overall pooled OR with 95% CI using random effects model. ISR, In-stent restenosis.

The pooled OR for ISR from the random effects model is shown in Figure 2. A total of 311 patients were used in this analysis (162 cases and 149 controls) (Table 2). No significant correlation was found between pioglitazone and the decrease in the rate of ISR (OR, 0.57; 95% CI, 0.24–1.34; P = 0.20), with low heterogeneity (I^2^ = 13.3%, P = 0.32). Furthermore, influential analysis showed that removal of any single trial did not essentially affected the overall pooled estimate. After excluding each trial in turn and recalculating the pooled estimates, these values and their significance was almost unchanged. We also performed sensitivity analyses to assess the robustness by examining the influence of various prespecified variables in included studies on the combined estimates for ISR as can be seen in Table 3. After exclusion of one study whose subjects were non-diabetes mellitus individuals or short follow-up (6 months), recalculation of OR yielded similar results (OR, 0.80, 95% CI, 0.32–1.98; P = 0.63), with no heterogeneity (I^2^ = 0.0%, P = 0.62). After exclusion of one study with low dose pioglitazone (<30 mg/d), the results were remained almost the same (OR, 0.43, 95% CI, 0.14–1.29; P = 0.13) with only low heterogeneity (I^2^ = 24.0%, P = 0.25).

**Table 3:** Sensitivity Analyses for ISR.

Outcomes	No. Trials	No. Patients*	OR(95% CI)	P value	P*_heterogeneity_* †	I^2^, %
Overal	3	162/149	0.57(0.24–1.34)	0.20	0.32	13.3
High dose (30 mg/d)	2	111/95	0.43(0.14–1.29)	0.13	0.25	24.0
Long follow-up (>6 months)	2	91/92	0.80(0.32–1.98)	0.63	0.62	0.0
T2DM	2	91/92	0.80(0.32–1.98)	0.63	0.62	0.0

CI, confidence interval; DM, Diabetes Mellitus; OR, Odds Ratios.

*The numerals indicate the total number of cases and controls.

† *P_heterogeneity_* less than 0.1 was considered significant.

### The Secondary Outcomes

Tables 4 outlines the results of follow-up angiography and IVUS. Late loss was less (WMD, −0.36; 95% CI, −0.64 to −0.07; P = 0.01) in pioglitazone-treated individuals by QCA analysis. However, pioglitazone was not associated with minimum lumen diameter (WMD, 0.30; 95% CI, −0.02 to 0.62; P = 0.06) and percentage stenosis of stented vessels (WMD, −6.30; 95% CI, −14.76 to 2.16; P = 0.63). Neointimal volume did not demonstrate significant difference (WMD, −0.73; 95% CI, −1.69 to 0.24; P = 0.63) between the pioglitazone-treated and control groups measured by IVUS.

**Table 4:** Clinical Endpoints: Continuous Data.

Outcomes	No. Trials	No. Patients*	WMD (95% CI)	P value	P*_heterogeneity_* †	I^2^, %
Minimum Lumen Diameter, mm	4	168/172	0.30 (−0.02 to 0.62)	0.06	0.00	85.0
Late Loss, mm	4	168/172	−0.36 (−0.64 to −0.07)	0.01	0.00	87.5
Percentage Stenosis, %	3	122/121	−6.30 (−14.76 to 2.16)	0.63	0.62	0.0
Neointimal Volume, mm^3^	2	91/92	−0.73(−1.69 to 0.24)	0.63	0.62	0.0

WMD, Weighted Mean Difference; CI, confidence interval.

*The numerals indicate the total number of cases and controls.

† *P_heterogeneity_* less than 0.1 was considered significant.

Among all 500 participants, there was 3 deaths in the control arm and no death in pioglitazone arm. TVR was lower in pioglitazone-treated patients (1.2% vs. 4.5%, OR: 0.25, 95% CI: 0.07 to 0.98, P = 0.04) compared with placebo-treated individuals. However, pioglitazone, as compared with controls, did not significantly reduce the incidence of TLR, MI, stent thrombosis and death as can be seen in Table 2.

### Publication Bias

The shape of the funnel plot was symmetrical, suggesting no significant publication bias. Further, the quantitative tests also did not show significant publication bias (Begg’s test, P = 0.602; Egger’s test, P = 0.650). It must be noted that the low power with only 5 studies included in our meta-analysis limited the interpretability of the finding.

## Discussion

As far as we know, the current meta-analysis of five RCTs including 255 pioglitazone-treated cases and 245 controls provides a first quantitative assessment of the possible impact of pioglitazone on ISR after DES implantation.

In this study, we have found that pioglitazone does not significantly reduce the incidence of ISR after DES implantation with low heterogeneity among the studies. However, we have also found that, when compared with control group, pioglitazone group shows significantly lower levels of late loss and TVR. In addition, for the other secondary outcomes, pioglitazone does not substantially affect the pooled estimates of these endpoints. The primary outcome of present meta-analysis is inconsistent with previous meta-analysis on prevention of ISR with pioglitazone. In detail, that meta-analysis [25] included studies whose individuals were treated with BMS implantation, whereas the present meta-analysis were treated with DES implantation. This main difference of study population may largely contribute to the discrepancy.

In order to obtain reliable results, only RCTs that clearly stated the inclusion criteria and patient characteristics were included in our meta-analysis. Moreover, our study have more sample size (involving 500 cases as opposed to 373 cases). In addition, we also performed multiple sensitivity analyses based on various prespecified variables to verify the robustness of our results. The ORs were not materially altered when we eliminated trials including non-diabetes mellitus individuals [26], [27], [28], with short follow-up (6 months) [26], [27], [28] or studies with low dose pioglitazone (15 mg/d) [26], [28], [29]. Furthermore, the influential analysis showed that removal of any single trial did not essentially affected the overall significance of ORs, which further confirm the robustness of the findings. However, Since only three of all five included studies [27], [29], [30] report related data of ISR, it was hard to reach a definitive conclusion based on limited sample data. Further studies are needed.

Previous studies have indicated that pioglitazone can reduce late loss, which is monotonically correlate with ISR risk. It is a representative and useful angiographic endpoint in stents studies [39]. Our meta-analysis lends support to prior work. However, other results of follow-up angiography and IVUS did not show any significant difference. Our meta-analysis have also showed that pioglitazone significantly reduces the risk of TVR, which is parallel to meta-analysis performed by Riche et al [40]. One meta-analysis [41] has shown that DES can reduce stent thrombosis compared with BMS. Disease duration, antiplatelet therapy discontinuation and stent number/length were the most common predictors of stent thrombosis [42]. In our meta-analysis, only one study performed by Lee et al. [29] has reported 3 cases of stent thrombosis, 1 patient in the pioglitazone group and 2 patients in the control group. Such a small sample size lacks power to reveal a significantly decreased risk. However, for the other clinical events, we failed to find significant differences.

The detailed mechanisms of restenosis have not yet been fully elucidated. The inflammatory response evoked by vascular damage during stent implantation is thought to be the main contributor to the development of restenosis [43]. Balloon dilation and stent placement during PCI lead to the endothelial denudation and subintimal hemorrhage, which initiates several proliferative processes, including neointimal hyperplasia, extracellular matrix formation, VSMCs proliferation and migration [44]. Previously preclinical [9], [11], [12] and clinical studies [30], [45] demonstrated that pioglitazone can exert its antiinflammatory, antiproliferative and antimigratory effect on all these processes. Pioglitazone can regulate some cellular and molecular parameters after stent implantation, these regulation include reduction in the number of monocyte and macrophage infiltration, circulating natural killer (NK) cells, decreased serum interleukin-6 (IL-6), matrix metalloproteinase (MMP)-1, MMP-9 and monocyte chemoattractant protein-1 (MCP-1) levels, and increased serum IL-10 concentration. Thus, these effects can inhibit migration and proliferation of VSMCs, neointimal hyperplasia and extracellular matrix formation during the vascular remodeling processes. In addition, pioglitazone enhances cytokine-mediated VSMCs apoptosis and further induced significant regression of intimal hyperplasia [46]. pioglitazone also prevents apoptosis of epithelial progenitor cells (EPCs) in mice as well as in human. Reduction of EPCs apoptosis may be a potentially beneficial mechanism for reduction of ISR [47].

Several limitations merit consideration in interpreting the findings and planning future studies. First, although we performed a comprehensive search of all eligible studies, only five studies with relatively small size met the inclusion criteria for this meta-analysis. The possibility of publication bias can not be completely excluded in meta-analysis, and this might potential distort the conclusion. Second, Much evidence indicate that genetic factors tend to increase the risk of restenosis, independent of conventional clinical parameters [48]. In our study, subjects predominantly related to Asian individuals, and different genetic background may lead to different results. Thus, further studies in other populations, such as Caucasian, will be needed to verify these results. Third, a great variability exists in the literature regarding timing, dosage, and duration of pioglitazone and further clarification and consistency for this is needed. Fourth, further studies should pay more attention to patients with special lesion characteristics such as long lesion length, calcified lesions, chronic total occlusions, and tortuous vessel. Because more­complex lesions tend to increase risk of ISR after DES implantation [49]. Fifth, Further subgroup analysis performed by other confounding factors such as gender, age, hypertension and smoking were unable to get from included trials. These factors have been regarded as effective variables for ISR. further studies should included these variables.

## Conclusions

The limited evidence indicates that pioglitazone does not demonstrate markedly beneficial effect in reducing ISR in patients subjected to coronary DES implantation, so pioglitazone should not be recommended for routine use currently. However, the results should be interpreted with care given the small sample size. Further large-scale RCTs are needed.

## Supporting Information

Checklist S1 **PRISMA 2009 checklist.**
(DOCX)
